# Clinical Presentation and Emergency Department Management Checkpoints of Acute Aortic Syndromes during the First Two Waves of the COVID-19 Pandemic

**DOI:** 10.3390/jcm12206601

**Published:** 2023-10-18

**Authors:** Paolo Bima, Jacopo Davide Giamello, Paolo Rubiolo, Francesca Risi, Paolo Balzaretti, Giuseppe Lauria, Domenico Vallino, Enrico Lupia, Fulvio Morello

**Affiliations:** 1 S.C. Medicina d’Urgenza U (MECAU), Ospedale Molinette, A.O.U. Città della Salute e della Scienza, 10126 Torino, Italy; paolo.bima@edu.unito.it (P.B.); enrico.lupia@unito.it (E.L.); 2 Dipartimento di Scienze Mediche, Università degli Studi di Torino, 10126 Torino, Italy; jacopo.giamello@edu.unito.it (J.D.G.);; 3 Cardiovascular Research Institute Basel (CRIB), 4056 Basel, Switzerland; 4 Medicina d’Urgenza, Ospedale S. Croce e Carle, 12100 Cuneo, Italy; 5 Dipartimento di Emergenza e Accettazione, Ospedale Mauriziano, 10128 Torino, Italy

**Keywords:** aorta, dissection, syndrome, COVID-19, SARS-CoV-2, emergency department

## Abstract

The COVID-19 pandemic has deeply affected the activity and patient flows of Emergency Departments (EDs), and concern for the worsening outcome of cardiovascular emergencies has been raised. However, the impact of COVID-19 on all subtypes of acute aortic syndromes (AASs) has not been evaluated so far. Cases of AASs managed in the ED of three hub hospitals in a large area of Northern Italy were retrospectively analyzed, comparing those registered during the pandemic (March 2020 to May 2021) with corresponding pre-COVID-19 periods. A total of 124 patients with AAS were managed during the COVID-19 period vs. 118 pre-COVID-19 (*p* = 0.70), despite a −34.6% change in ED visits. Posterior chest pain at presentation was the only clinical variable with a different prevalence (46.0% vs. 32.2%, *p* = 0.03). Surgery and endovascular treatment rates were unchanged. Time intervals influenced by patient transfer to the hub center were longer during the COVID-19 period and longest during high viral circulation periods. Ninety-day mortality was unchanged, with a higher mortality trend during the pandemic surges. In conclusion, ED presentation and care of AASs were marginally affected by COVID-19, but efforts are needed to preserve efficient patient transfer to specialized centers and prevent mortality, especially during pandemic peaks.

## 1. Introduction

Acute aortic syndromes (AASs) are a group of deadly conditions involving the thoracic aorta and sharing common pathophysiological, clinical, and therapeutic features. They include aortic dissection (AD), intramural aortic hematoma (IMH), penetrating aortic ulcer (PAU), and spontaneous aortic rupture (SAR) [1]. AASs are relatively rare, affecting 2 to 15 cases/100,000 individuals/year, but their unspecific clinical presentation includes common symptoms such as chest, back, abdominal pain, syncope, and neurological deficits [2]. Key factors affecting clinical outcomes of AASs are rapid diagnosis and treatment, with Emergency Medical Services and Departments (EDs) acting as key players in the initial management and transfer of patients from spoke to specialized aortic centers [3,4].

The COVID-19 pandemic has caused rapid saturation of health care resources for non-COVID-19 patients, with a negative impact on elective interventions for AAS risk conditions (aortic aneurysms and aortic valve diseases) and on blood pressure control [5,6,7]. The pandemic has also heavily impacted patient flows, case mix, clinical activity, and the organization of EDs [8]. Initial spreading of COVID-19 was associated with an unprecedented drop in the number of total ED visits and in the number of patients diagnosed with acute cardiovascular conditions, with a possible subsequent rebound in related hospitalizations [9,10,11,12,13]. Furthermore, COVID-19 has emerged as a novel risk factor for cardiovascular emergencies involving immunothrombosis [14]. A study evaluating the Society of Thoracic Surgeons Adult Cardiac Surgery Database found similar outcomes in patients with type A AD treated before and during the first surge of the pandemic [15]. However, another study found that COVID-19 positivity worsened perioperative outcomes in patients with type A AD [16]. To our knowledge, the effects of the COVID-19 pandemic on other AAS subtypes and the focus on pre-surgical data are lacking.

In this study, we sought to evaluate the characteristics of ED presentation, management, and outcomes of AASs during the COVID-19 era in consecutive patients presenting or transferred to one of three EDs functioning as hub centers for AASs in a reasonably large geographic area of Italy. Results are relevant to inform and update acute cardiovascular care and emergency medicine practice in the current endemic COVID-19 phase and potentially during new infectious outbreaks.

## 2. Materials and Methods

### 2.1. Setting

This retrospective observational study was performed in the high-volume EDs located in the 3 AAS referral centers treating patients in the Western Piedmont and Valle d’Aosta areas of Northern Italy: Ospedale Molinette (Torino), Ospedale Mauriziano (Torino), and Ospedale S. Croce e Carle (Cuneo). In 2019, the census of the population living in the referral area was 2.97 million subjects. The study complied with the Declaration of Helsinki, and the local Ethics Committee (Comitato Etico Interaziendale A.O.U. Città della Salute e della Scienza di Torino–A.O. Ordine Mauriziano–A.S.L. Città di Torino) approved the study (00042/2020), and patients provided written informed consent and oral consent (the latter during the COVID-19 period, due to infection risk management in the ED).

### 2.2. Inclusion and Exclusion Criteria

Inclusion criteria were: (1) patient age ≥ 18 years; (2) evaluation in the ED for acute symptoms, dating no more than 14 days; and (3) confirmed diagnosis of AAS. The following disease entities were all considered AASs: AD, IMH, PAU, and SAR, classified as either type A or B according to the Stanford classification. Exclusion criteria include: (1) not datable aortic disease, including asymptomatic or occasional findings during work-up for non-related conditions; and (2) chronic aortic syndromes (i.e., symptom onset > 14 days).

### 2.3. Patient Search and Inclusion Process

Patients were identified and annotated prospectively during the ED visit and, secondly, by query on the electronic ED database using ICD-9-CM codes identifying AASs (Supplementary Table S1), in order to obtain consecutive AAS patients managed in the study periods. ED charts (including the chart of the spoke ED referring patient) and hospital charts were collected and examined for satisfaction of inclusion and exclusion criteria. Cases with incomplete clinical charts were excluded. 

**Figure 1:**
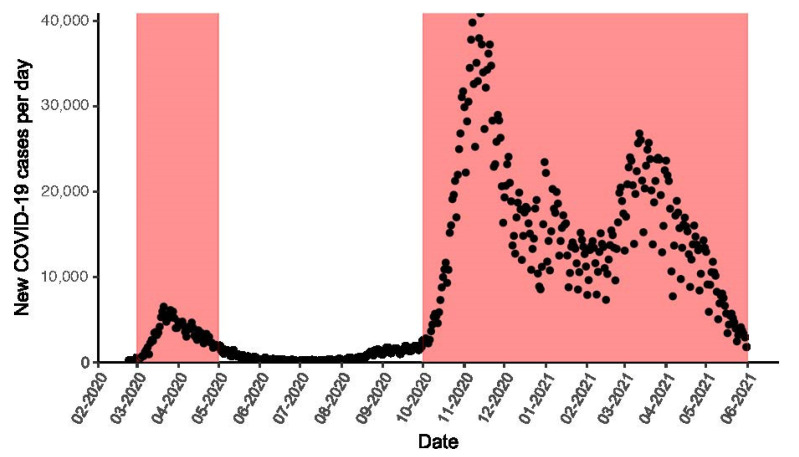
Number of new COVID-19 cases per day in Italy. Red-shaded areas represent pandemic peaks and high viral circulation periods.

For data analysis, two time periods were identified: the COVID-19 period (from 1 March 2020 to 31 May 2021) and the pre-COVID-19 period (from 1 March 2018 to 31 May 2018 and from 1 March 2019 to 29 February 2020). The COVID-19 period was chosen based on the local detection of the first COVID-19 clusters on 21 February 2020, with national lockdown measures imposed from 9 March 2020, until the end of the second pandemic wave (Figure 1). The pre-COVID-19 period was chosen to coincide with the same months of the COVID-19 period, considering the seasonality of AAS incidence [17].

### 2.4. Collected Variables and Outcomes

For each patient, the following variables were obtained from the medical charts and inputted in an anonymized database: gender, age, presenting symptoms, symptom onset, past medical history, clinical variables defining pre-test probability of AAS, vital parameters at presentation, laboratory values (white blood cell count, creatinine, D-dimer, troponin), nasopharyngeal swab for SARS-CoV-2 at ED presentation and during hospitalization, date/time of triage (first ED contact and hub center if the patient was transferred), date/time of CTA (or other advanced imaging) leading to diagnosis, AAS type, date/time of surgery or endovascular repair (EVAR), survival/death during hospitalization and at 90 days. The pre-test probability of AAS was measured using the aortic dissection detection (ADD) risk score and the simplified AORTAs score (Supplementary Tables S2 and S3) [1,18].

Outcomes compared between the COVID-19 and pre-COVID-19 periods were: number of AASs, intervals between key clinical timestamps, and mortality. 

### 2.5. Statistical Analysis

General characteristics were assessed with median and interquartile range (IQR) for continuous variables and with proportion and 95% confidence interval (CI) for categorical variables. Statistical differences were compared using the Mann–Whitney U test for continuous variables and the χ^2^ or Fisher’s exact test for proportions, as appropriate. Count data were expressed as an absolute number and proportion. 

Count data were compared with the Poisson regression, through which the percent (%) change and its CI were estimated from the exponentiated Poisson regression coefficient. Time differences were compared using a multivariate linear regression model for the natural logarithm (ln) of the time differences, with the COVID-19 period as the reference period and “center” as an additional independent variable. The percent change was calculated from the exponentiated beta coefficient. Differences in 90-day mortality were assessed using a multivariate Cox regression model using the pre-COVID-19 period as the reference period and type A AD as the reference for AAS subtypes, after adjusting for age, sex, and nasopharyngeal swab positivity. The Kaplan–Meier estimator was used to plot the survival curves of each AAS subtype, divided into pre-COVD-19 and COVID-19 periods. The survival curves were compared using the log-rank test. A sensitivity analysis grouping patients presenting during low-viral circulation timeframes with patients presenting in the pre-COVID-19 period was carried out to evaluate the impact of different COVID-19 pandemic phases on time differences and 90 day mortality. *p*-values were considered significant if <0.05. Statistical analysis was carried out using R version 4.1.3 (R Foundation for Statistical Computing, Vienna, Austria). 

## 3. Results

### 3.1. Characteristics of AAS Patients in the Two Periods

The census of total ED visits in the participating centers was 154 517 in the COVID-19 period and 236 318 in the pre-COVID-19 period (percent change −34.6% [95% CI −34.8 to −34.4], *p* < 0.001). In the study period, 266 patients were selected as potential cases of AASs; 24 patients were excluded for having a final diagnosis different from an AAS (13 from the pre-COVID-19 period and 11 from the COVID-19 period), and 242 patients were further analyzed (Supplementary Figure S1). A total of 124 were managed during the COVID-19 period and 118 during the pre-COVID-19 period (*p* = 0.70 for % change), corresponding to an incidence of 3.34 and 3.18 cases/100.000 ED visits/year, respectively (rate ratio 1.05 [95% CI 0.81–1.36], *p* = 0.70). A total of 87 (70.2%) patients presented to a spoke ED during the COVID-19 period vs. 81 (68.6%) during the pre-COVID-19 period (*p* = 0.80). 

Table 1 shows the demographic and clinical data of patients with AASs in the COVID-19 period, compared to the pre-COVID-19 period. There was no significant difference for most variables except for posterior chest pain at presentation, which was more frequent in the COVID-19 period (46.0% vs. 32.2%, *p* = 0.03). In the COVID-19 period, four patients tested positive for SARS-CoV-2, of whom one died within 90 days.

### 3.2. AAS Subtypes and Treatment Strategies

**Figure 2:**
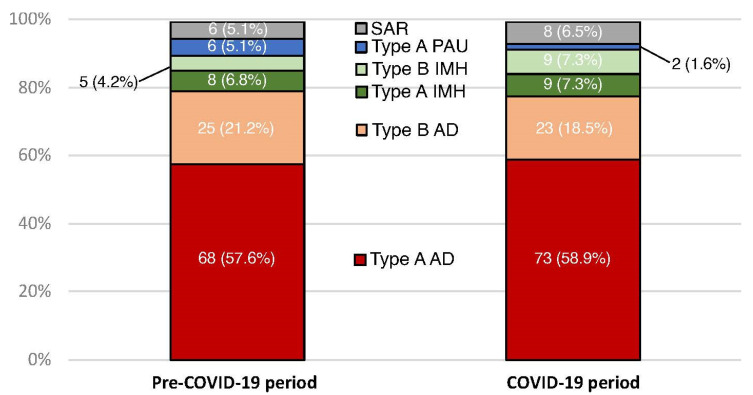
Cumulative bar plot showing AAS subtypes according to pre-COVID-19 or COVID-19 study periods. Numbers are shown as counts (percentage). AD: aortic dissection; IMH: intramural hematoma; PAU: penetrating aortic ulcer; SAR: spontaneous aortic rupture.

AAS subtypes managed during the COVID-19 and pre-COVID-19 periods are shown in Figure 2. There was no statistical difference in the % change of different AAS subtypes (Table 2). 

In the COVID-19 period, 83 (66.9%) patients underwent surgery and 19 (15.3%) thoracic endovascular aortic repair (TEVAR), vs. 84 (71.2%, *p* = 0.3) and 10 (8.5%; *p* = 0.10), respectively, in the pre-COVID-19 period. Among the seventy-three patients with type A AD (for whom surgery is the gold standard treatment) in the COVID-19 period, eight (11.0%) were not operated on (in the pre-COVID-19 period, they were 6/68 [8.8%], *p* = 0.89). These eight patients were significantly older (median age 75 [67 to 84] vs. 65 [55 to 77] years, *p* = 0.03) and comorbid (CAD frequency was 38% vs. 7.7%, *p* = 0.04). The time from symptom onset to ED presentation was 150 (60 to 240) minutes in patients who underwent surgery and 360 (225 to 900) minutes in patients who did not undergo surgery (*p* = 0.02). This was not true for the pre-COVID-19 period, when only the age was significantly higher in patients who did not undergo surgery.

### 3.3. Time Intervals

Time intervals between management checkpoints are shown in Table 3. Intervals influenced by patient transfer from spoke to hub center were longer during the COVID-19 period: triage at spoke to triage at hub ED 296 vs. 248 min (*p* = 0.008) in the pre-COVID-19 period, and triage at spoke to surgery 312 vs. 263 min (*p* = 0.04). These findings remained consistent in the sensitivity analysis grouping patients presenting during the pre-COVID-19 period with pandemic periods at low viral circulation: triage at spoke to triage at hub ED 297 vs. 251 min (*p* = 0.10), and triage at spoke to surgery 313 vs. 283 min (*p* = 0.03), as shown in supplementary Table S4. The other time intervals (time from symptom onset to ED presentation, time from first triage to CTA, time at hub ED to surgery for transferred patients) were comparable between the two periods and in the sensitivity analysis. 

### 3.4. Outcome

**Figure 3:**
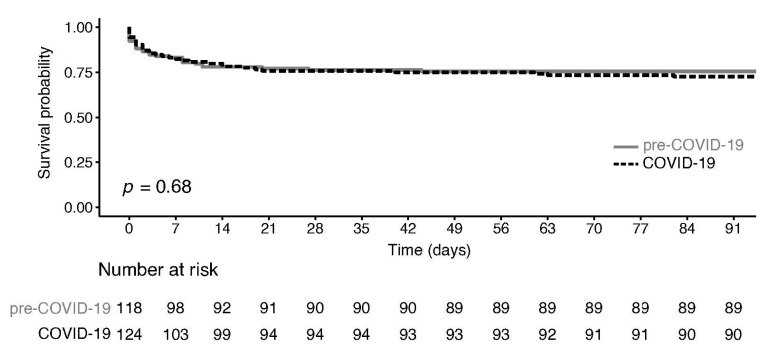
Kaplan–Meier estimator of 90-day mortality stratified according to pre-COVID-19 or COVID-19 periods.

Patient stratification according to 90-day mortality is shown in supplementary Table S5. As expected, non-survivors were older, presented more frequently with hypotension or shock, had lower BP values, higher hs-cTn blood levels, and were more frequently diagnosed with SAR. Overall all-cause 90-day mortality was 27.4% in the COVID-19 period and 24.6% in the pre-COVID-19 period (*p* = 0.68 at the log-rank test, Figure 3). Across the whole study, 90-day mortality for type A AD it was 27.0%, for type B AD it was 12.5%, for type A IMH it was 23.5%, for type B IMH it was 14.3%, for type A PAU it was 12.5%, and for SAR it was 85.7%. All were not statistically different in the two study periods (Supplementary Figure S2A–F). In a multivariate Cox-regression model adjusted for age, the COVID-19 period had an HR of 1.09 (0.66–1.79) for 90-day mortality (Supplementary Table S6). In the sensitivity analysis, the HR for mortality during the high viral circulation period was 1.64 (0.98–2.75, *p* = 0.06). A positive COVID-19 test was not a statistically significant predictor of 90-day mortality in both analyses, although the number of positive NF swabs was small (N = 4).

## 4. Discussion

To our knowledge, this is the first study assessing the characteristics of ED presentation, management, and outcome characteristics of all AAS subtypes during the COVID-19 pandemic, as well as estimating the effect of high vs. low viral circulation pandemic phases. Based on data regarding other cardiovascular emergencies such as myocardial infarction and stroke and preliminary reports on small case series, the COVID-19 pandemic has been postulated as a potential detrimental factor for the diagnosis and management of AASs in Emergency Medical Services and EDs, leading to worse clinical outcomes [9,10,11,12,13,16,19]. Data from our study detected a potential detrimental effect of COVID-19 on AAS mortality only during high viral circulation periods.

A key finding of our study is that the total number of AASs and subtypes diagnosed in the COVID-19 period was not significantly different from the pre-COVID-19 period, despite a major decrease in total ED visits. This data suggests the absence of any detectable effect of COVID-19 on the risk factors and pathological/molecular mechanisms leading to AASs. The stable number of AASs managed in the COVID-19 period indicates that the hypothesis of missed AAS cases due to underuse of ED services by patients during the COVID-19 pandemic is unlikely [10,11,20]. However, the design of the study does not provide insights on the incidence of AASs during the COVID-19 period, as the number of AASs presenting with out-of-hospital sudden death could potentially be different in the two periods. Also, the time from symptom onset to triage was unchanged in the COVID-19 period, further indicating that the COVID-19 pandemic did not lead to increased latency in hospital evaluation or to the late presentation of patients with AASs. Furthermore, the percentage of patients transferred from spokes to hub centers was similar in the two study periods, reassuring the local preservation of the AAS transfer process to hub centers during the COVID-19 pandemic. 

We found that presentation signs and symptoms were not different in the two periods, except for posterior chest pain, which was reported more frequently in the COVID-19 period. This was not associated with an increased frequency of Stanford type B subtypes. The cause of this finding is unknown. A possible explanation could be the increased attention given to treating physicians for posterior chest pain during the COVID-19 period due to the potential correlation of this symptom with the pulmonary and pleural consequences of COVID-19. However, this data needs confirmation in other case series. An important finding is that patient stratification according to pre-test risk scores (ADD, AORTAs) was not modified in the COVID-19 period, indicating that their applicability is not modified throughout pandemic periods.

This study did not find statistically significant delays in the presentation to the ED and diagnosis of AAS with CTA, although it did show an increase in time intervals involving patient transfer from spoke to aortic hub center, reaching their longest delays during high viral circulation periods. In contrast, this study did not show any delay in hub centers for surgery access. This could be due to the saturation of ambulance services for COVID-19 patients, with an increased number of transports per day and an increased delay for vehicle disinfection [21,22]. Surgery and TEVAR procedures were unchanged during the two periods, suggesting that the reorganization of EDs, radiology, cardiac, and vascular surgery units did not significantly affect the potential of aortic centers. This finding is in line with a previous report of UK surgery services during COVID-19 in terms of access to surgery and turn-down proportion [23]. However, a delay in ED presentation could not be ruled out as a factor affecting surgery turn-down, although older age and comorbidities may have a larger role in this decision, as “late presenters” had their median symptom onset (only) 6 h prior to presentation. 

Overall mortality for AASs was similar in the COVID-19 vs. pre-COVID-19 period in our cohort, in line with a previous study from China [24]. Although the prevalence of COVID-19 NF swab positivity was low in our cohort, a positive COVID-19 NF swab was not a significant predictor for mortality, differently from a previous study involving only perioperative outcomes of patients with type A AD [16]. Apart from the different outcomes considered in the two studies, the present study could be underpowered to detect the impact of swab positivity on 90-day mortality. Mortality by AAS subtype was also comparable to pre-COVID-19 standards [25]. Nonetheless, this study showed a trend towards higher mortality during the high viral circulation periods, possibly and partly related to the higher transfer times. Taken together, these results seem to indicate that, while the hospital services reorganization, including surgical and interventional radiology departments, could efficiently cope with the COVID-19 pandemic, ambulance transfer services suffered most during the pandemic surges, possibly reflecting the overwhelming request of ambulances for pre-hospital emergency care. 

Some limitations must be highlighted. First, data collection in a retrospective study, notwithstanding the author’s efforts, could be imprecise. Second, the study may be unsuited for subgroup comparisons or the detection of small changes. Third, these findings might not be generalizable to other healthcare systems. However, most countries suffered similar restrictions during the pandemic, and Northern Italy was one of the most heavily struck areas in the world by the first and second waves of COVID-19 [26].

In conclusion, we found that in a large representative area of Northern Italy, despite significant diversion of staff and system resources, ED presentation of AASs was largely unchanged, and only checkpoints involving patient transfer were affected by the rise of the COVID-19 pandemic. In clinical terms, the most relevant finding was a reduced efficiency of patient transfer from spoke to hub centers, warranting specific corrections in case of future pandemic outbursts, and a trend towards higher mortality during pandemic surges.

## Figures and Tables

**Table 1:** General characteristics of the patients stratified according to pre-COVID-19 and COVID-19 periods.

Characteristic	Overall (N = 242)	Pre-COVID-19 Period (N = 118)	COVID-19 Period (N = 124)	*p*-Value
**Demographics**				
Age (years)	71 (58, 78)	71 (61, 80)	70 (57, 78)	0.4
Sex (Female)	78 (32.2%)	35 (29.7%)	43 (34.7%)	0.4
Symptoms onset (hours)	3.0 (2.0, 10.0)	3.0 (1.1, 9.8)	3.0 (2.0, 12.0)	0.3
**Presenting signs and symptoms**				
Anterior chest pain	145 (59.9%)	68 (57.6%)	77 (62.1%)	0.5
Posterior chest pain	95 (39.3%)	38 (32.2%)	57 (46.0%)	0.03
Abdominal pain	75 (31.0%)	38 (32.2%)	37 (29.8%)	0.7
Lumbar pain	33 (13.6%)	12 (10.2%)	21 (16.9%)	0.13
Limb ischemia	18 (7.4%)	11 (9.3%)	7 (5.6%)	0.3
Syncope	40 (16.5%)	17 (14.4%)	23 (18.5%)	0.4
Hypotension	48 (19.8%)	23 (19.5%)	25 (20.2%)	0.9
Hypotension, shock, or cardiac arrest	52 (23.5%)	26 (24.8%)	26 (22.4%)	0.7
**Comorbidities**				
Hypertension	154 (63.6%)	75 (63.6%)	79 (63.7%)	0.9
Diabetes	16 (6.6%)	6 (5.1%)	10 (8.1%)	0.4
Active smoking	64 (26.4%)	31 (26.3%)	33 (26.6%)	0.9
Coronary artery disease	24 (9.9%)	10 (8.5%)	14 (11.3%)	0.5
**Risk factors for AAS**				
Connective tissue disease	7 (2.9%)	2 (1.7%)	5 (4.0%)	0.4
Known TAA	40 (16.5%)	17 (14.4%)	23 (18.5%)	0.4
Aortic valve disease	12 (5.0%)	5 (4.2%)	7 (5.6%)	0.6
Family history of AAS	9 (3.7%)	3 (2.5%)	6 (4.8%)	0.5
Recent aortic manipulaton (<1 month)	10 (4.1%)	4 (3.4%)	6 (4.8%)	0.7
**Pain characteristics**				
Abrupt onset	154 (63.6%)	72 (61.0%)	82 (66.1%)	0.4
Severe pain (NRS = 7)	170 (70.2%)	81 (68.6%)	89 (71.8%)	0.6
Ripping pain	98 (40.5%)	45 (38.1%)	53 (42.7%)	0.5
**High-risk features at the physical examination**				
Perfusion deficit	63 (26.0%)	29 (24.6%)	34 (27.4%)	0.6
Neurological deficit	42 (17.4%)	21 (17.8%)	21 (16.9%)	0.9
New diastolic aortic murmur	3 (1.2%)	3 (2.5%)	0 (0.0%)	0.11
**Risk scores**				
ADD score				0.5
0	24 (9.9%)	14 (11.9%)	10 (8.1%)	
1	97 (40.1%)	50 (42.4%)	47 (37.9%)	
2	97 (40.1%)	45 (38.1%)	52 (41.9%)	
3	24 (9.9%)	9 (7.6%)	15 (12.1%)	
High risk per ADD score (≥2)	121 (50.0%)	54 (45.8%)	67 (54.0%)	0.2
High clinical probability per AORTAs (≥2)	165 (74.7%)	75 (71.4%)	90 (77.6%)	0.3
**Vital signs and selected biomarkers at presentation**				
SBP (mmHg)	130 (105, 150)	130 (100, 153)	130 (110, 150)	0.9
DBP (mmHg)	75 (60, 85)	80 (60, 90)	75 (60, 84)	0.3
HR (bpm)	78 (65, 90)	78 (64, 90)	79 (67, 90)	0.6
White blood cells (10^9^/L)	12.3 (9.2, 16.8)	12.2 (8.8, 18.7)	12.3 (9.6, 15.8)	0.9
Creatinine (mg/dL)	1.0 (0.8, 1.3)	1.0 (0.8, 1.3)	1.0 (0.8, 1.4)	0.5
hs-cTn (ng/L)	14.5 (7.2, 38.0)	16.0 (7.3, 50.0)	14.0 (7.4, 30.5)	0.7
D-dimer (mg/dL)	5126 (1723, 20,000)	4629 (1780, 20,000)	5419 (1540, 19,974)	0.9

ADD: Aortic Dissection Detection, DBP: diastolic blood pressure, HR: heart rate, hs-cTn: high-sensitivity cardiac troponin, NRS: numeric rating scale, SBP: systolic blood pressure, TAA: thoracic aortic aneurysm.

**Table 2:** Percent change and its 95% CI for the total number of AAS and their subtypes in Poisson regression models adjusted for hub center. AAS: Acute Aortic Syndrome; AD: aortic dissection; IMH: intramural hematoma; PAU: penetrating aortic ulcer; SAR: spontaneous aortic rupture.

Dependent Variable	Percent Change (95% CI)	*p*-Value
All AASs	5.1% (−18.3–35.3)	0.70
Type A AD	7.4% (−22.9–49.6)	0.67
Type B AD	−8.0% (−48.1–62.3)	0.77
Type A IMH	12.5% (−57.0–199.5)	0.81
Type B IMH	80.0% (−37.8–486)	0.29
PAU	−66.6% (−96.2–33.6)	0.18
SAR	33.4% (−53.7–305)	0.59

**Table 3:** Time intervals between major checkpoints. The percent change was calculated with a linear regression model adjusted for age and AAS subtype, with the natural logarithm of the time interval as the dependent variable.

Time Interval (min)	COVID-19 Period	Pre-COVID-19 Period	Percent Change (95% CI)	*p*-Value
Symptom onset to first ED triage	180 (120, 720)	120 (60, 525)	34.6% (−10.4–102)	0.15
Triage to the CTA	115 (52, 238)	97 (53, 229)	13.0% (−18.1–55.9)	0.46
First triage to surgery	264 (170, 450)	217 (146, 454)	12.4% (−13.9–46.7)	0.39
Triage at spoke ED to surgery	312 (206, 560)	263 (163, 478)	32.7% (2.0–72.6)	0.04
Triage at the hub ED to surgery (only transferred patients)	30 (12, 54)	27 (9, 41)	18.1% (−27.3–91.9)	0.50
Triage at spoke ED to triage at hub ED	296 (187, 530)	248 (132, 369)	45.1% (10.3–90.8)	0.008

CTA: CT angiography, ED: emergency department.

## Data Availability

Data are made available upon reasonable request to the corresponding author (Fulvio Morello).

## Supplementary Materials

The following supporting information can be downloaded at: https://www.mdpi.com/article/10.3390/jcm12206601/s1, Table S1. ICD-9-CM codes used to retrieve patients from the electronic health records; Table S2. The ADD risk score; Table S3. The AORTAs risk score. The total score is obtained by summing the points of each risk factor; Table S4. Sensitivity analysis of time intervals between major checkpoints, according to high or low viral circulation, which was coupled with the pre-COVID period. The percent change was calculated with a linear regression model adjusted for age and AAS subtype with the natural logarithm of the time interval as the dependent variable; Table S5. Patient baseline characteristics by 90-day mortality; Table S6. Multivariable Cox regression model for 90-day mortality. The regression model was adjusted for age and sex. 4 NF swabs were positive, of whom 1 died within 90-day; Figure S1. Patient flowchart; Figure S2. Kaplan-Meier estimator of 90-day mortality for type A AD (A), type B AD (B), type A IMH (C), type B IMH (D), type A PAU (E), SAR (F).
