# Supplementary material for: Clinical Presentation and Emergency Department Management Checkpoints of Acute Aortic Syndromes during the First Two Waves of the COVID-19 Pandemic
Source: J Clin Med. 2023 Oct 18;12(20):6601. doi: 10.3390/jcm12206601 (PMC10607079; doi:10.3390/jcm12206601)
Supplement: Supplementary file 1 [file jcm-12-06601-s001.zip › jcm-2632374-supplementary.pdf]

Clinical presentation and Emergency Department management checkpoints of acute aortic syndromes during the first two waves of COVID-19 pandemic

SUPPLEMENTARY TABLES AND FIGURES

Table S1. ICD-9-CM codes used to retrieve patients from the electronic health records.

Table S2. The ADD risk score.

Table S3. The AORTAs risk score. The total score is obtained by summing the points of each risk factor.

Table S4. Sensitivity analysis of time intervals between major checkpoints, according to high or low viral circulation, which was coupled with the pre-COVID period. The percent change was calculated with a linear regression model adjusted for age and AAS subtype with the natural logarithm of the time interval as the dependent variable.

Table S5. Patient baseline characteristics by 90-day mortality.

Table S6. Multivariable Cox regression model for 90-day mortality. The regression model was adjusted for age and sex. 4 NF swabs were positive, of whom 1 died within 90-day.

Figure S1. Patient flowchart.

Figure S2. Kaplan-Meier estimator of 90-day mortality for type A AD (A), type B AD (B), type A IMH (C), type B IMH (D), type A PAU (E), SAR (F)

Table S1. ICD-9-CM codes used to retrieve patients from the electronic health records.

ICD-9-CM code	Description
441.0	Aortic dissection
441.00	Dissection of aorta, unspecified site
441.01	Dissection of aorta, thoracic
441.02	Dissection of aorta, abdominal
441.03	Dissection of aorta, thoracoabdominal
441.1	Thoracic aneurysm, ruptured
441.2	Thoracic aneurysm without mention of rupture
441.5	Aortic aneurysm of unspecified site, ruptured
441.6	Thoracoabdominal aneurysm, ruptured
441.7	Thoracoabdominal aneurysm, without mention of rupture
441.9	Aortic aneurysm of unspecified site without mention of rupture

Table S2. The ADD risk score.

High-risk conditions	High-risk chest pain features	High-risk signs
• Marfan syndrome or other connective tissue disease<br>• Family history of aortic disease<br>• Known aortic valve disease<br>• Recent aortic manipulation<br>• Known thoracic aortic aneurysm	Chest, back, or abdominal pain described as:<br>• Abrupt in onset<br>• Severe in intensity<br>• Ripping or tearing in quality	• Pulse deficit or systolic blood pressure differential<br>• Focal neurologic deficit (with pain)<br>• Murmur of aortic insufficiency (new, with pain)<br>• Hypotension or shock state

Table S3. The AORTAs risk score. The total score is obtained by summing the points of each risk factor.

Item	Points
Hypotension/shock	2
Thoracic aortic aneurysm	1
Severe pain	1
Sudden pain	1
Pulse deficit	1
Neurologic deficit (with pain)	1

Table S4. Sensitivity analysis of time intervals between major checkpoints, according to high or low viral circulation, which was coupled with the pre-COVID period. The percent change was calculated with a linear regression model adjusted for age and AAS subtype with the natural logarithm of the time interval as the dependent variable.

Time interval (minutes)	High viral circulation period (N = 79)	Pre-COVID/ low viral circulation period (N = 163)	Percent change (95% CI)	*P*-value
symptom onset to first ED triage	180 (60, 480)	180 (120, 600)	-9.2% (-41.1 — 40.5)	0.66
triage to CTA	119 (51, 257)	99 (54, 227)	11.1% (-20.6 — 55.2)	0.54
first triage to surgery	247 (172, 507)	245 (145, 432)	23.9% (-6.8 — 64.5)	0.14
triage at spoke ED to surgery	313 (202, 590)	283 (175, 473)	34.5% (2.2 — 77.1)	0.03
triage at hub ED to surgery (only transferred patients)	23 (7, 38)	34 (11, 52)	17.4% (-49.9 — 44.8)	0.55
triage at spoke ED to triage at hub ED	297 (199, 495)	251 (144, 396)	27.0% (-4.9% — 69.8)	0.10

Table S5. Patient baseline characteristics by 90-day mortality.

Characteristic	Overall (N = 242)	Alive (N = 179)	Dead at 90 days (N = 63)	P-value
**Demographics**				
Age (years)	71.0 (58.4, 78.0)	69.9 (56.0, 77.3)	76.0 (66.4, 83.4)	<0.001
Sex (Female)	78 (32.2%)	54 (30.2%)	24 (38.1%)	0.2
Symptoms onset (hours)	3.0 (2.0, 10.0)	3.0 (2.0, 10.4)	2.0 (1.0, 10.0)	0.2
**Presenting signs and symptoms**				
Anterior chest pain	145 (59.9%)	107 (59.8%)	38 (60.3%)	0.9
Posterior chest pain	95 (39.3%)	77 (43.0%)	18 (28.6%)	0.04
Abdominal pain	75 (31.0%)	58 (32.4%)	17 (27.0%)	0.4
Lumbar pain	33 (13.6%)	24 (13.4%)	9 (14.3%)	0.9
Limb ischemia	18 (7.4%)	11 (6.1%)	7 (11.1%)	0.3
Syncope	40 (16.5%)	25 (14.0%)	15 (23.8%)	0.07
Hypotension	48 (19.8%)	28 (15.6%)	20 (31.7%)	0.006
Hypotension, shock or cardiac arrest	52 (23.5%)	29 (17.5%)	23 (41.8%)	<0.001
**Comorbidities**				
Hypertension	154 (63.6%)	110 (61.5%)	44 (69.8%)	0.2
Diabetes	16 (6.6%)	9 (5.0%)	7 (11.1%)	0.14
Active smoking	64 (26.4%)	56 (31.3%)	8 (12.7%)	0.004
Coronary artery disease	24 (9.9%)	17 (9.5%)	7 (11.1%)	0.7
**Risk factors for AAS**				
Connective tissue disease	7 (2.9%)	5 (2.8%)	2 (3.2%)	0.9
Known TAA	40 (16.5%)	30 (16.8%)	10 (15.9%)	0.9
Aortic valve disease	12 (5.0%)	6 (3.4%)	6 (9.5%)	0.08
Family history of AAS	9 (3.7%)	6 (3.4%)	3 (4.8%)	0.7
Recent aortic manipulaton (<1 month)	10 (4.1%)	8 (4.5%)	2 (3.2%)	0.9
**Pain characteristics**				
Abrupt onset	154 (63.6%)	112 (62.6%)	42 (66.7%)	0.6
Severe pain (NRS ≥7)	170 (70.2%)	124 (69.3%)	46 (73.0%)	0.6
Ripping pain	98 (40.5%)	73 (40.8%)	25 (39.7%)	0.9
**High-risk features at the physical examination**				
Perfusion deficit	63 (26.0%)	42 (23.5%)	21 (33.3%)	0.12
Neurological deficit	42 (17.4%)	32 (17.9%)	10 (15.9%)	0.7
New diastolic aortic murmur	3 (1.2%)	1 (0.6%)	2 (3.2%)	0.2
**Risk scores**				
ADD score				0.4
0	24 (9.9%)	19 (10.6%)	5 (7.9%)	
1	97 (40.1%)	75 (41.9%)	22 (34.9%)	
2	97 (40.1%)	70 (39.1%)	27 (42.9%)	
3	24 (9.9%)	15 (8.4%)	9 (14.3%)	

High risk per ADD score	121 (50.0%)	85 (47.5%)	36 (57.1%)	0.2
AORTAs - high clinical probability	165 (74.7%)	120 (72.3%)	45 (81.8%)	0.2
**Vital signs and selected biomarkers at presentation**				
SBP (mmHg)	130.0 (105.0, 150.0)	130.0 (110.0, 155.0)	120.0 (91.2, 140.0)	0.009
DBP (mmHg)	75.0 (60.0, 85.0)	80.0 (65.0, 90.0)	70.0 (60.0, 80.0)	0.006
HR (bpm)	78.0 (65.0, 90.0)	77.0 (65.0, 89.0)	85.0 (66.5, 96.5)	0.2
White blood cells (10^9/L)	12.3 (9.2, 16.8)	11.5 (9.1, 15.8)	13.8 (9.9, 18.7)	0.04
Creatinine (mg/dL)	1.0 (0.8, 1.3)	1.0 (0.8, 1.3)	1.1 (0.8, 1.5)	0.10
hs-cTn (ng/L)	14.5 (7.2, 38.0)	14.0 (7.2, 28.0)	29.0 (8.5, 91.5)	0.04
D-dimer (mg/dL)	5,126.0 (1,723.0, 20,000.0)	4,417.5 (1,432.8, 17,420.8)	7,244.5 (2,865.2, 28,146.0)	0.15
**AAS subtype**				
Type A AD	141 (58.3%)	103 (57.5%)	38 (60.3%)	0.7
Type B AD	48 (19.8%)	42 (23.5%)	6 (9.5%)	0.02
Type A IMH	17 (7.0%)	13 (7.3%)	4 (6.3%)	0.9
Type B IMH	14 (5.8%)	12 (6.7%)	2 (3.2%)	0.5
PAU	8 (3.3%)	7 (3.9%)	1 (1.6%)	0.7
SAR	14 (5.8%)	2 (1.1%)	12 (19.0%)	<0.001
**Treatment**				
Surgery	167 (69.0%)	129 (72.1%)	38 (60.3%)	0.08
TEVAR	19 (7.9%)	15 (8.4%)	4 (6.3%)	0.8
Medical therapy	58 (24.0%)	37 (20.7%)	21 (33.3%)	0.04

AD: aortic dissection, ADD: Aortic Dissection Detection, DBP: diastolic blood pressure, EVAR: endovascular aortic repair, HR: heart rate, hs-cTn: high-sensitivity cardiac troponin, IMH: intramural hematoma, NRS: numeric rating scale, PAU: penetrating aortic ulcer, SAR: spontaneous aortic rupture, SBP: systolic blood pressure TAA: thoracic aortic aneurysm.

Table S6. Multivariable Cox regression model for 90-day mortality. The regression model was adjusted for age and sex. 4 NF swabs were positive, of whom 1 died within 90-day.

Variable	Hazard Ratio (95% CI)	*P*-value
COVID period	1.09 (0.66 – 1.79)	0.74
Positivity to SARS-CoV2 NF swab	0.80 (0.11 – 5.95)	0.83
AAS subtype		
Type A AD	1.0 (reference)	-
Type B AD	0.37 (0.15 – 0.90)	0.03
Type A IMH	0.57 (0.20 – 1.62)	0.29
Type B IMH	0.39 (0.09 – 1.64)	0.20
Type A PAU	0.30 (0.04 – 2.26)	0.24
SAR	4.03 (2.05 – 7.93)	<0.001

AD: aortic dissection, IMH: intramural hematoma, NF: nasopharyngeal, PAU: penetrating aortic ulcer, SAR: spontaneous aortic rupture

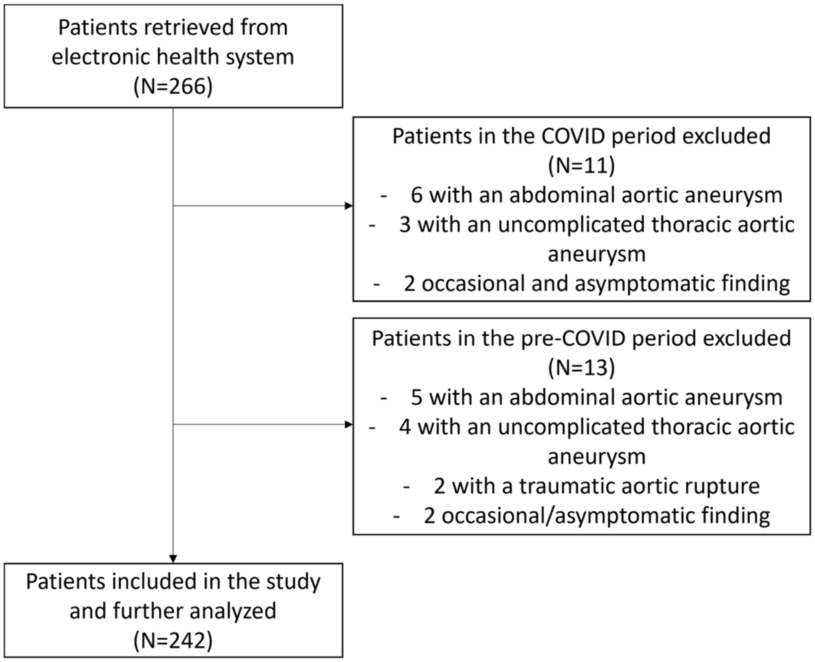

Figure S1. Patient flowchart.

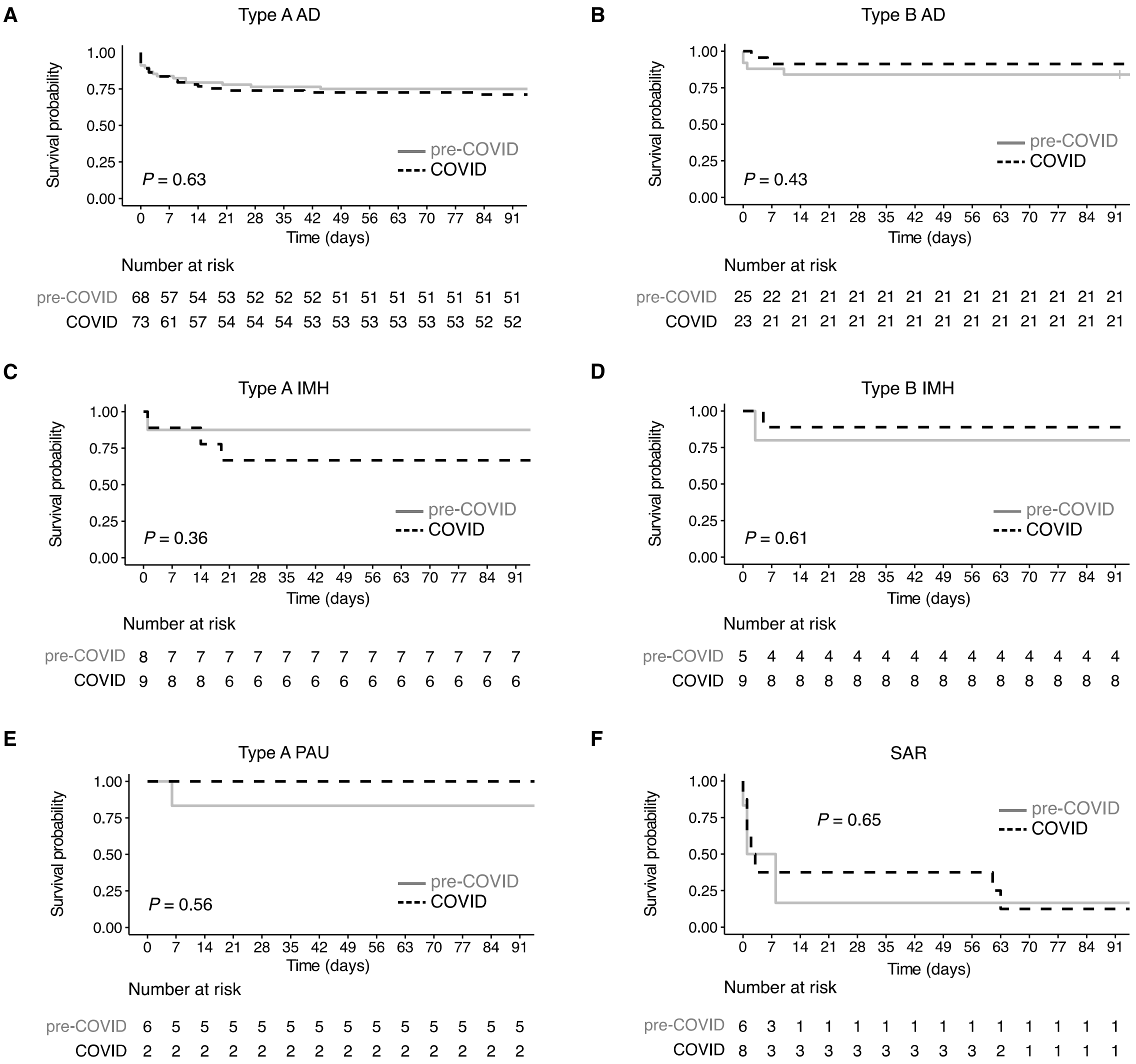

Figure S2. Kaplan-Meier estimator of 90-day mortality for type A AD (A), type B AD (B), type A IMH (C), type B IMH (D), type A PAU (E), SAR (F).
